# Insights from the 2-year-long human confinement experiment in Grand Cayman reveal the resilience of coral reef fish communities

**DOI:** 10.1038/s41598-023-49221-y

**Published:** 2023-12-09

**Authors:** Jack V. Johnson, Alex D. Chequer, Gretchen Goodbody-Gringley

**Affiliations:** Reef Ecology and Evolution Lab, Central Caribbean Marine Institute, Little Cayman, Cayman Islands

**Keywords:** Marine biology, Ecology, Community ecology

## Abstract

In March 2020, the world went into lockdown to curb the spread of the novel coronavirus (SARS-CoV-2), with immediate impacts on wildlife across ecosystems. The strict 2-year long lockdown in Grand Cayman provided an unprecedented opportunity to assess how the ‘human confinement experiment’ influenced the community composition of reef fish. Using a suite of multivariate statistics, our findings revealed a stark increase in reef fish biomass during the 2 years of lockdown, especially among herbivores, including parrotfish, with drastic increases in juvenile parrotfishes identified. Additionally, when comparing baseline data of the community from 2018 to the 2 years during lockdown, over a three-fold significant increase in mean reef fish biomass was observed, with a clear shift in community composition. Our findings provide unique insights into the resilience of reef fish communities when local anthropogenic stressors are removed for an unprecedented length of time. Given the functional role of herbivores including parrotfish, our results suggest that reductions in human water-based activities have positive implications for coral reef ecosystems and should be considered in future management strategies.

## Introduction

Coral reef fishes are a salient group of organisms for reef habitats, contributing to their ecosystem function and the provision of key ecosystem services, including nutrient cycling^1^, herbivory to prevent macroalgae proliferation over coral^2^, and cascading trophic effects^3,4^, among others. These functions promote coral reef resilience^5^ and drive key ecosystem services such as nutrient provision from fisheries^6^, storm protection^7^, and other economic benefits for human livelihoods^8–10^. As such, reef fishes are crucial for overall health of coral reefs, and potentially the wellbeing of 443 million people who live within 30 km of a coral reef^11^. However, owing to anthropogenic activity, coral reefs are highly vulnerable and considered one of the most endangered ecosystems in the Anthropocene^12,13^. This puts reef fish populations at risk, as they face numerous local and global stressors that jeopardize their survival^14,15^.

Anthropogenic exploitation of coral reefs at the local scale often impacts reef fish communities through activities that directly remove fish from the environment such as overfishing^16,17^, destructive fishing practises^18,19^, and overharvesting of aquaria species^20^. Additionally, pollution from boats in the form of spillages and noise can reduce fitness^21,22^, lead to mortality^22,23^, and hence influence population viability, ultimately affecting reef fish biomass and community composition. Understanding alterations to reef fish community composition is crucial, as many reef fish species are documented to provide critical roles in maintaining algal overgrowth and cycling nutrients that assist in sustaining a coral-dominated benthic community, with some herbivores serving as keystone species on coral reefs^1,2,24^. Reductions in biomass, abundance, and species richness may therefore alter overall coral reef health^5,24,25^, while changes in behaviour that influence the local biomass of herbivores can alter the benthic dynamics (i.e. patchiness) within marine systems^26^.

During early 2020, the COVID-19 pandemic sent 4.5 billion humans into lockdown^27^ with the sudden cessation of anthropogenic activity known as the Anthropause^28^, presenting the opportunity to assess how wildlife responds to the “human confinement experiment”^27^. During this time, direct reductions in sea-based activities were observed around the globe, with notable decreases in shipping traffic^29^ associated with anthropogenic noise^22,30^ and pollution^31^. Pollution in the form of anthropogenic noise and chemical pollution from sea vessels negatively impacts fish behaviour^31–33^ and physiology^31,33,34^ thereby influencing community composition. During the short period when tourism and heavy water-based activities were reduced between March and May 2020, coral reef ecosystems in several regions that typically experience high anthropogenetic pressures showed immediate increases in fish species diversity and abundance^35–37^, linked to increased recruitment of juvenile fishes^38^. While marked increases immediately following a short respite is positive, studies examining how longer lockdown restrictions on water-based activities influenced the community composition are lacking.

**Figure 1:**
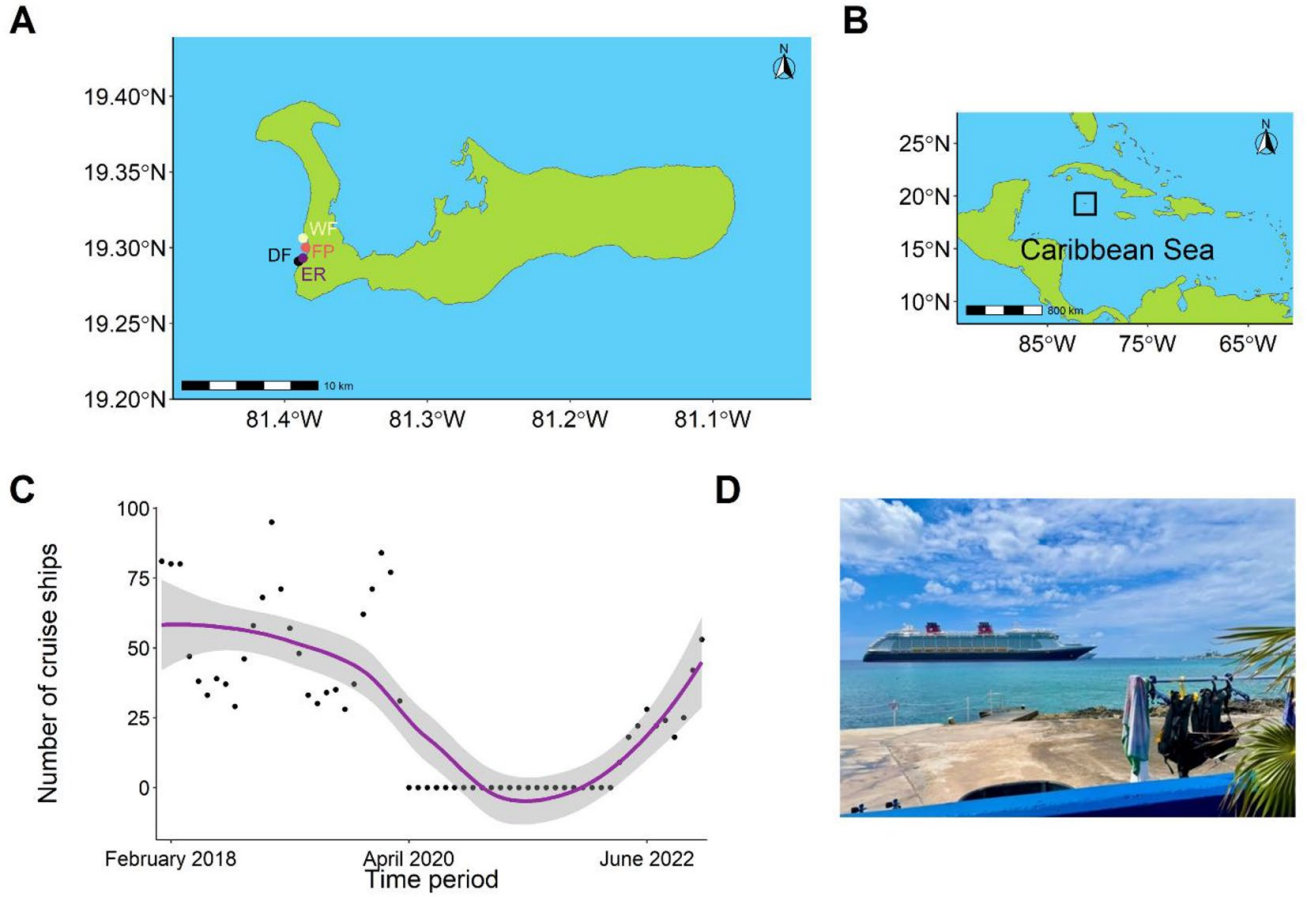
Location of the survey sites in Grand Cayman (**A**) (*DF* Don Fosters, *ER* Edens Rock, *FP* Fish Point, *WF* Wharf) shown within the Caribbean Sea (**B**). The black box in (**B**) indicates the location of Grand Cayman. (**C**) is the number of large passenger vessels visiting Grand Cayman form January 2018 through to December 2022 with a trendline (purple line) fit using locally weighted scatterplot smoothing showing 95% confidence intervals (grey area). (**D**) View from Eden Rock survey sites of cruise ships in port on April 26, 2022 (photo by GG-G).

The unique COVID-19 lockdown restrictions on water-based activities in Grand Cayman (Fig. 1A,B) that extended for nearly 2 years provided an unparalleled opportunity^28^ to assess how the reef fish community responds when sea-based anthropogenic activity are reduced for a significant period of time (Table 1, Fig. 1C). Here, we took advantage of this opportunity by periodically surveying reef fish communities near the main harbour of Grand Cayman, from July 2020 to June 2022, which spanned the duration of lockdown restriction and beyond the reopening of the island to tourism. Using a suite of multivariate statistical approaches, we document the response of fish communities to this period of altered activity, which provides unprecedented insights to the impacts of anthropogenetic activity and the resilience of reef fish populations.

**Table 1:** Brief timeline of restrictions to water-based activities in Grand Cayman.

Key dates	Restrictions	Source
24-Mar-20	Full lockdown in effect. All sea-based activities including scuba diving, wildlife tours, cruises, and fishing are prohibited	Cayman Island Government Hazard Management department. https://www.exploregov.ky/covid19-timeline-cayman
15-May-20	Boating permitted for the purpose of fishing only	
16-May-20	Beaches are open for exercise	
07-Jun-20	Beaches and leisure boating are permitted with no restrictions. This is only available to residents of Grand Cayman. No cruise ships or large passenger vessels are allowed to enter Grand Cayman waters	
21-Mar-22	First cruise ships return to Grand Cayman	Cayman Island Port Authority. https://www.caymanport.com/ship-schedules-calendar/

## Results

### Patterns of fish species richness, biomass, and abundance

**Figure 2:**
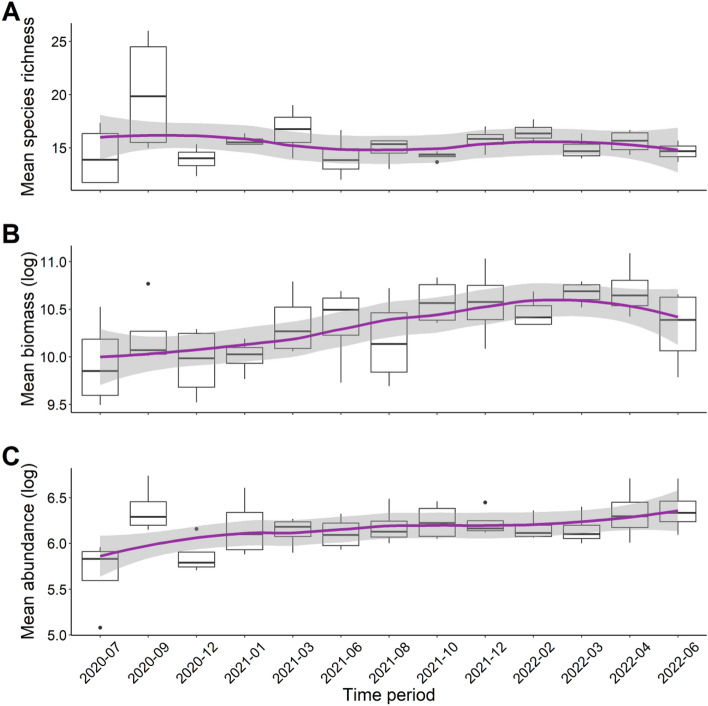
Overall trends in (**A**) species richness (**B**), biomass (**C**), and abundance of all fish across the study period. Boxes represent the first and third interquartile, whiskers show the range of the data calculated as 1.5 times the interquartile, horizontal bar represent the medium, and dots indicate outliers. The trend line is fitted using locally weighted scatterplot smoothing with 95% confidence intervals (grey area).

Overall, mean species richness varied from a low of 11.7 at Eden Rock in July 2020 to a high of 26 at the same site, Eden Rock, in September 2020 (Fig. 2A). Mean biomass was lowest in July 2020 at 13.28(± 0.53) kg, and highest in April 2022 at 65.35(± 3.2) kg (Fig. 2B), while the mean abundance of all fish during a survey month varied from a low of 160(± 4) in July 2020 to a high of 844(± 14) in September 2020 in Fig. 2C.

### Fish response to time

**Figure 3:**
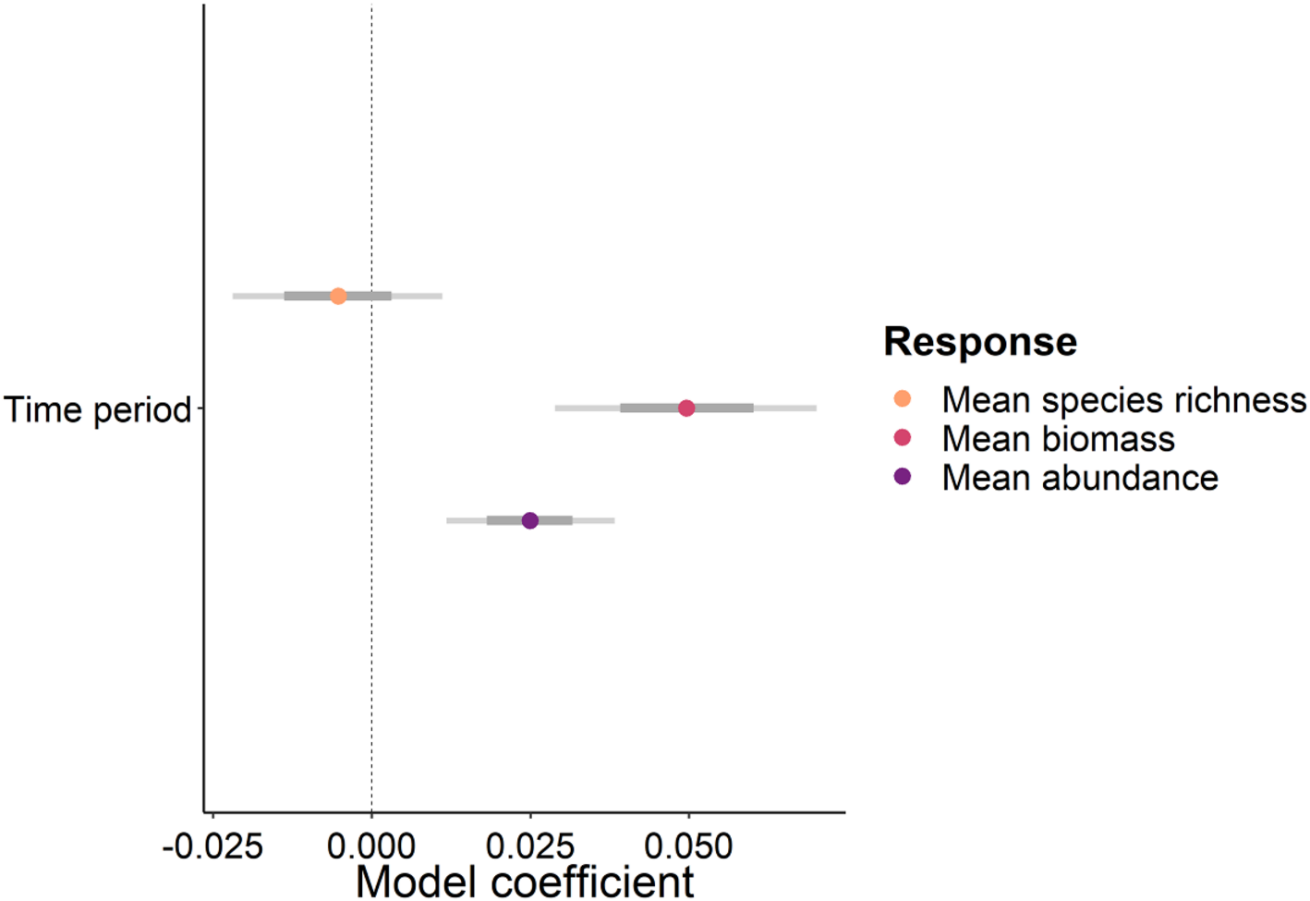
Coefficient estimates from the random effect multivariate Bayesian model showing the predictions of species richness, biomass, and abundance in response to time. Points are coloured to indicate the response variable. Thick horizontal grey bars show the 80 credible intervals (CI), while thin grey bars show the 95% CI. Dashed vertical line indicates zero. An effect is considered to exist if the 95% CIs do not cross zero. The time-period (y-axis) are the rescaled dates from July 2020 to June 2022.

Our Bayesian model reveals a high probability that the overall biomass and abundance of fishes increased over time after the introduction of restrictions to water-based activities (Fig. 3). However, no probability of an increase or decrease in species richness was observed.

When modelling each trophic guild individually, only herbivores (all herbivorous fish species) showed a high probability of both increased biomass and abundance over time (Table 2). Meanwhile, omnivore biomass and planktivore abundance were the only other measures showing a credible increase over the study period (Table 2). No trophic guilds elicited any probable decrease in species richness, biomass, or abundance since the introduction of COVID 19 restrictions to water-based activities (Table 2).

**Table 2:** Coefficient estimates from the multivariate random effect Bayesian model showing the response of species richness, biomass, and abundance throughout the study period for each trophic guild.

Model	Trophic group	Response	Estimate	Lower 95% CI	Upper 95% CI
Time (rescaled month and year)	Herbivore	Richness	− 0.02	− 0.06	0.03
		**Biomass**	**0.06**	**0.01**	**0.04**
		**Abundance**	**0.05**	**0.03**	**0.06**
	Invertivore	Richness	0	− 0.03	0.04
		Biomass	0	− 0.03	0.04
		Abundance	− 0.01	− 0.04	0.01
	Macrocarnivore	Richness	− 0.02	− 0.07	0.03
		Biomass	0.03	− 0.03	0.08
		Abundance	0	− 0.04	0.04
	Omnivore	Richness	− 0.02	− 0.06	0.01
		**Biomass**	**0.06**	**0.02**	**0.1**
		Abundance	0.01	− 0.01	0.03
	Planktivore	Richness	0	− 0.07	0.07
		Biomass	0	− 0.03	0.04
		**Abundance**	**0.06**	**0.04**	**0.09**

Probability relationships where the credible intervals (CI) do not cross zero are highlighted in bold.

**Figure 4:**
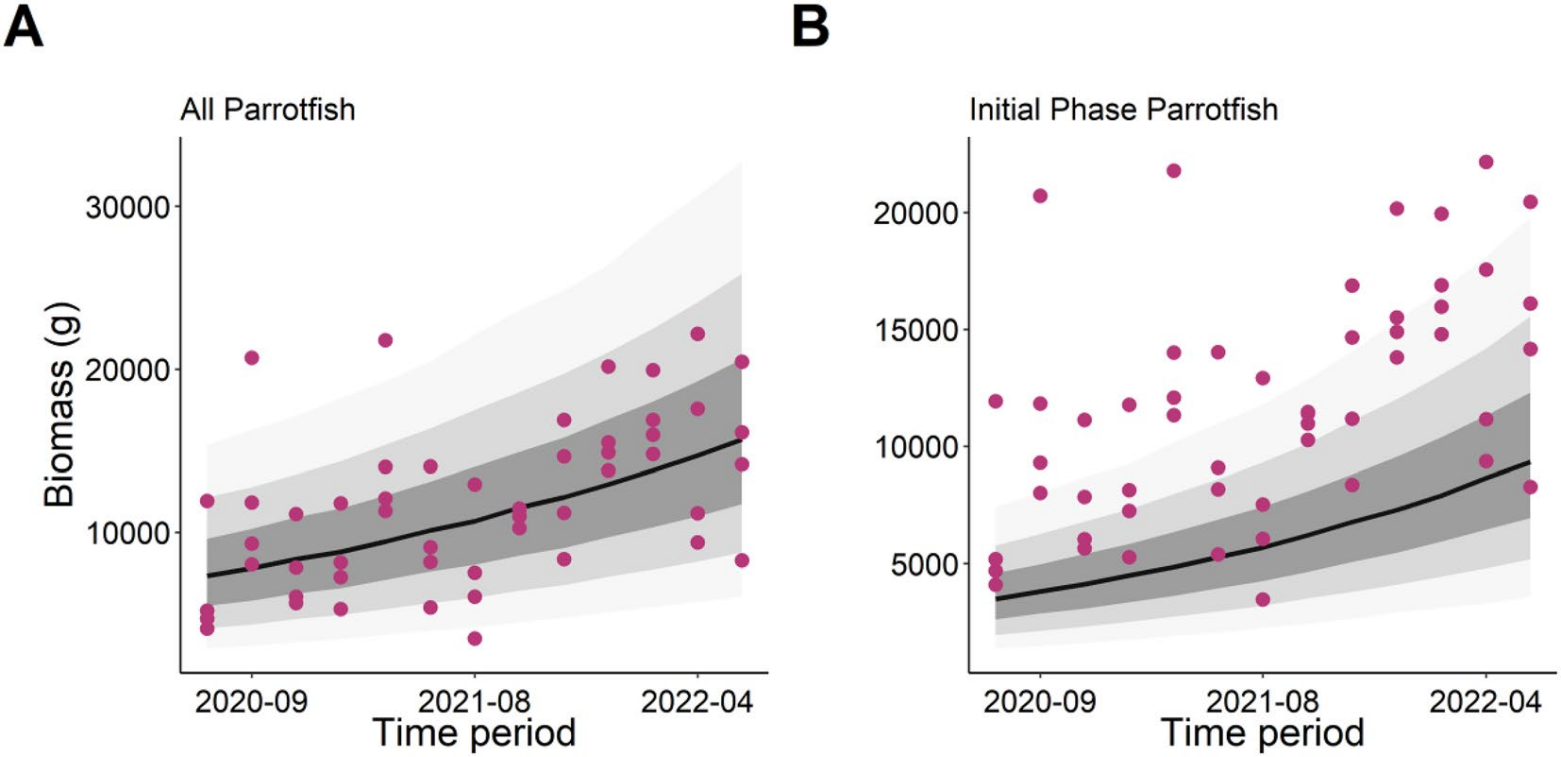
Predictions from the random effect Bayesian model showing the response of (**A**) all Parrotfish and (**B**) initial phase Parrotfish biomass over the study period. Pink points represent the raw data input into the model with dark grey bars showing 50% credible intervals (CI), medium grey bars showing 80% CI, and light grey bars showing 95% CI. Parrotfish biomass was strongly predicted to increase over the study period for all Parrotfish (β = 0.06, u95 = 0.09, l95 = 0.04) and initial phase Parrotfish (β = 0.08, u95 = 0.11, l95 = 0.05).

Parrotfish biomass showed a high probability of increasing over the study period (Fig. 4A), with juvenile Parrotfish showing an even higher probability of increasing since the introduction of restrictions which prevented water-based activities (Fig. 4B).

### Shifts in community composition before, during, and after lockdown

**Figure 5:**
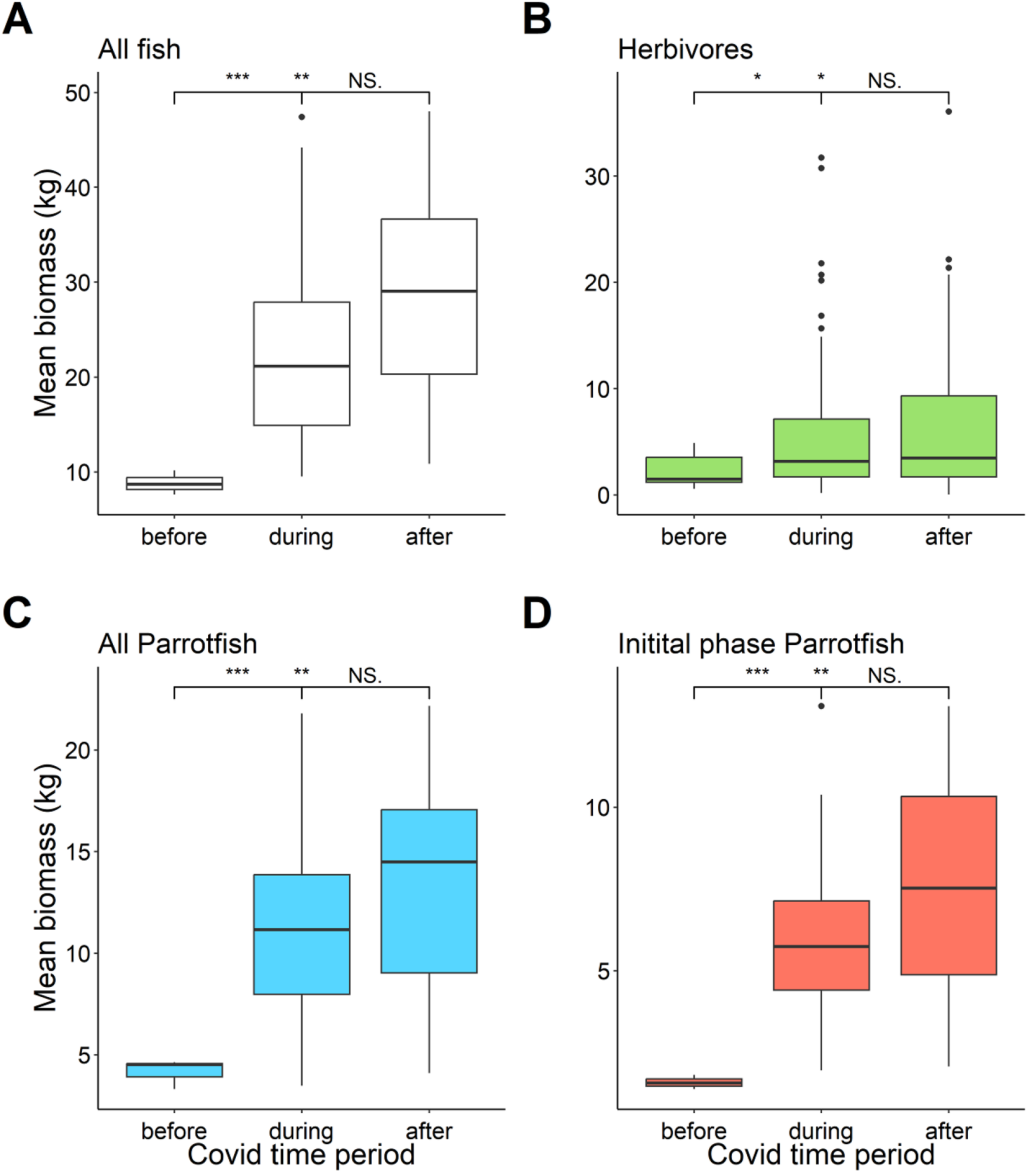
Boxplots of reef fish biomass before COVID lockdown (baseline data from July 2018), during lockdown, and after the reopening of the Cayman Islands to large passenger vessels. (**A**) The biomass of all fish, (**B**) just herbivores, (**C**) all parrotfish, and (**D**) juvenile parrotfish. Boxes represent the first and third interquartile, whiskers show the range of the data calculated as 1.5 times the interquartile, horizontal bar represent the medium, and dots indicate outliers. Colours are from the Stoplight parrotfish *Sparisoma viride* based on hex-codes extracted from the fishualize^65^ package. Significance tests were plotted using the ggsignif^66^ package with p-value significant levels plotted as *** = 0.001, ** = 0.01, and * = 0.05.

Quantifying the biomass of reef fish in Grand Cayman before lockdown compared to during lockdown (Fig. 5), significant increases in biomass was observed for all fish species (Z = − 2.577, Adj. P = 0.02), all herbivorous species (Z = − 2.452, Adj. P = 0.028), all phases of parrotfish species (Z = − 2.496, Adj. P = 0.025) and initial phase Parrotfishes only (Z = − 2.626, Adj. P = 0.017). No significant differences in fish biomass were found for the period during lockdown compared to the period immediately following the return of cruise ships (Fig. 5).

**Figure 6:**
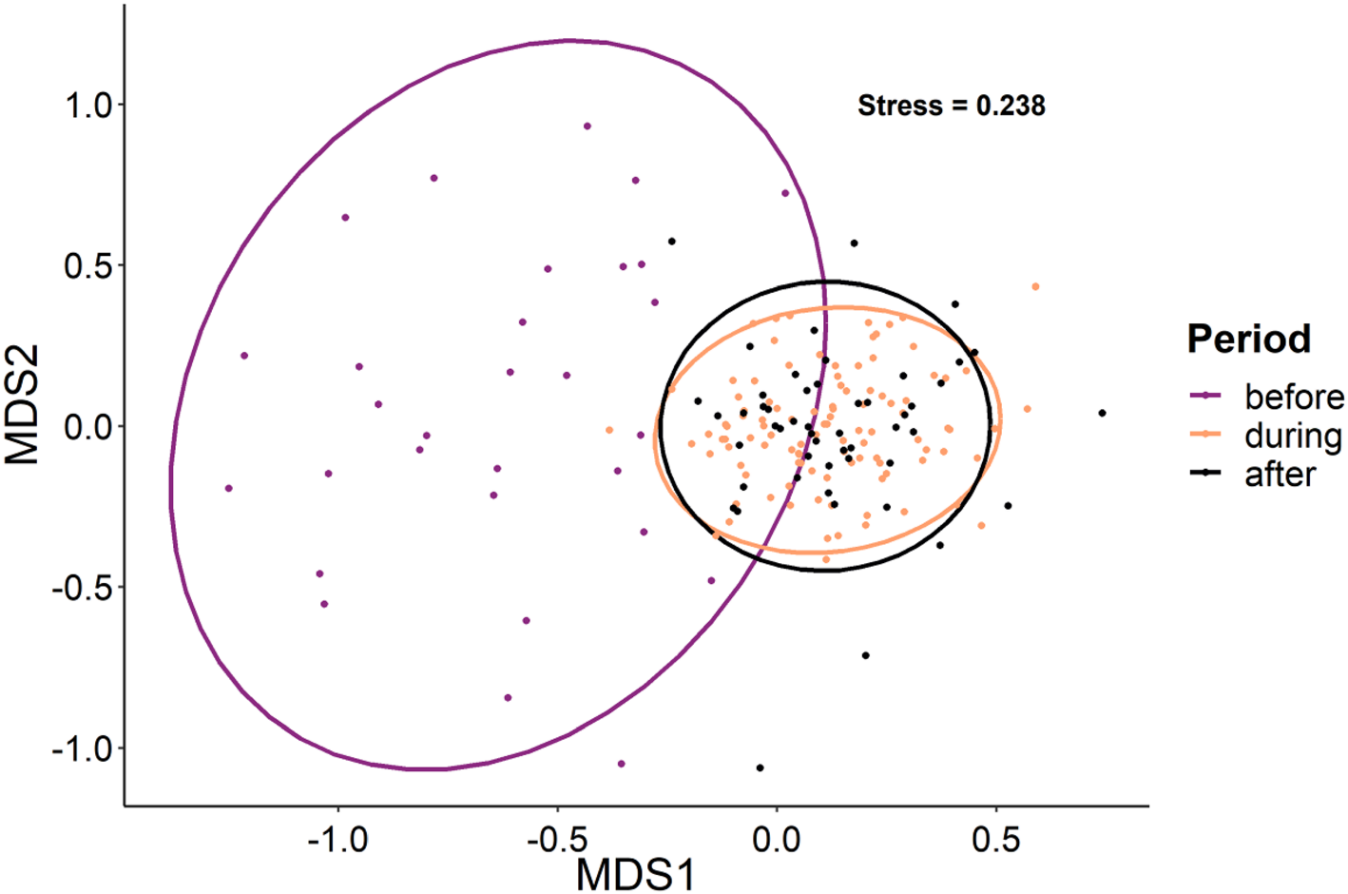
Ordinations of reef fish communities in Grand Cayman visualised using non-metric multidimensional scaling. Points show each fish survey for each time-period with standard deviation-based ellipses encompassing 95% of the data points in each group.

The community composition of reef fishes in Grand Cayman shifted significantly from before the COVID lockdown compared to during and after lockdown (Fig. 6, PERMANOVA, df = 2, R^2^ = 0.313, F = 41.446, P < 0.001). A post-hoc pairwise analysis revealed a strong significant difference in the fish community before, compared to during (df = 1, R2 = 0.362, F = 74.952, P = 0.003) and after lockdown (df = 1, R2 = 0.347, F = 42.039. P = 0.003), while a weak significant difference was observed in the community during vs after lockdown (df = 1, R2 = 0.015, F = 2.371, P = 0.012). An overview of changes in the abundance and biomass of reef fish species can be found in Table S2 and Fig. S9.

## Discussion

Our findings document increased biomass and abundance of coral reef fish populations during the extent of the COVID-19 lockdown restrictions implemented from March 2020 to February 2022 for Grand Cayman and suggest that continued increases as a result of the lockdown are highly probable (Fig. 3, Table 2). Notably, herbivorous species showed increases in both fish biomass and abundance, likely driving the overall trends observed. Among the herbivorous fish groups, parrotfish, and juvenile parrotfish in particular, showed significant increases in biomass, which will likely have positive consequences for reef ecosystem functioning. Remarkably, the three-fold increase in fish biomass after lockdown compared to before lockdown highlights the remarkable resilience of reef fish, with the alteration of the entire community likely having consequences for reef function and resilience.

The observed increases in fish biomass throughout the period of lockdown restrictions in Grand Cayman can most likely be attributed to two main reasons: (1) behavioural shifts impacting habitat distribution^26^; and (2) improved fitness leading to increased recruitment. Firstly, cessation of water-based activities would result in reduced ambient sound and physical disturbance in the water leading to immediate changes in the behaviour of fishes that will likely become bolder and less cryptic, thereby encouraging redistribution and habitation of areas previously saturated by boats and people^35–38^. Additionally, reductions in stressful conditions caused by water-based activities, such as noise and waste from large boats, also impacts key behaviours including feeding, sociality, reproduction, and subsequently, physiology^21–23,31–33^. Essentially, the immediate rise in fish biomass and abundance is likely a combination of increased boldness of cryptic fish, and new habitation of previously highly impacted environments by reef fish. Secondly, these changes in behaviour resulting from reduced stress likely increased ecological fitness of reef fish^22,32,39^ leading to improved reproduction and recruitment, as seen with the clear increase in initial phase parrotfish biomass (Fig. 5B). Whether the increase in juvenile fish biomass observed in our study was due to increased reproductive success or a redistribution of juvenile fishes cannot be determined in the present study, however, our unique 2-year data set provides sufficient temporal coverage to suggest that increased recruitment was related to reduced activity associated with the COVID lockdown. However, it should be noted that only three sampling periods after the resumption of normal shipping activity were conducted. Resampling these sites over a longer time-period would be highly insightful into understanding the longer-term consequences of strong management on water-based activities for effecting the fish community. Ultimately, the long-term increases in reef fish biomass are likely a result of both changes in fish behaviour and improved ecological fitness and regardless of the cause will aid in the long-term stability of these populations.

Moreover, increases in biomass of key functional groups during this period of quiescence has important consequences for the ecosystem function of coral reefs and their derived ecosystem services. For example, parrotfishes are the dominant herbivores on modern day Caribbean coral reefs and are suggested to maintain ecosystem stasis by preventing algal overgrowth that results in secondary decreases in coral recruitment^3,5,40^. Thus, the increases in parrotfish biomass observed here will likely to be beneficial for sustaining a coral dominated reef ecosystem. Consequently, our findings highlight how restrictions to water-based activities can lead to increased biomass of reef fish populations, suggesting that the implementation of restrictions through management strategies, such as Marine Protected Areas (MPAs), can be effective tools for enhancing fish populations^13,25,41,42^. In turn, higher fish biomass of key functional groups contributes to the ecosystem function of coral reefs^5,43^ and the provision of ecosystem services^5,8,9^. However, it should be noted that the benefits from reduced anthropogenic activity will not enhance reef resilience under climate change^13,44–46^, as isolation and restrictions do not confer enhanced resistance or recovery to corals from global warming^45–48^. Rather, these findings indicate fish biomass can return to reefs quickly when restrictions to water-based activities are enacted, and that biomass can increase because of enhanced fitness encouraging higher levels of fish recruitment. Such increases will help to ensure provision of key ecosystem services^9^ and may enhance coral reef resilience to disturbance events^5,13,25,49^. By focusing on key groups of fish such as herbivores, including parrotfish, we show that management of anthropogenic activity on sea based activities can increase fish biomass, which in turn could have consequences for ecosystem health^13^.

In summary, lockdown restrictions on water-based activities in response to COVID-19 are associated with increased biomass of coral reef fish, particularly parrotfish and juvenile parrotfish. Given the clear ubiquitous impacts of COVID-19 lockdowns around the world on behaviour and physiology of multiple organisms across multiple ecosystems^27,28^, increased biomass of reef fish is not surprising^35–38^. However, our study highlights the effect over a longer time-period (2 years) than previous studies^35–37^ (~ 3 months), indicating increased biomass was likely driven by both altered behaviour and increased ecological fitness owing to lockdown restrictions, which subsequently contributed to fish recruitment. Our findings show how tight restrictions to water-based activities can positively influence biomass of functionally important reef fish, which could have consequences for coral reef ecosystem function and coral reef health in the Anthropocene.

## Methods

### Study sites and data collection

Data were collected from four sites in Grand Cayman (Fig. 1) with surveys conducted from a range of 10.5–43 ft (median = 27 ft). The first temporal point of surveying took place in July 2020 where surveys were conducted every other month through June 2022. For each site, fish point transects (n = 3–5 per sampling period per site) were conducted within the above depth range depending on haphazard selection of transect location using 30 m by 2 m transects with every fish counted identified to species levels. Each fish was categorized into size classes based on total length (0–5 cm, 6–10 cm, 11–20 cm, 21–30 cm, 31–40 cm, and > 40 cm) to allow for biomass calculations using the formula:

$$W=a*L^b$$

where W is the weight of the fish, *L* is the maximum length based on the size classes above. *a* and *b* are species specific constants based on empirical data for calculating fish biomass from size-weight relationships^50–53^. These constants were obtained from fish base, with values from congenic species used if data for a specific species were not available^54^. Each fish species was subsequently grouped into the appropriate trophic guild based with grouping also derived from Fishbase^54^. Fish where we were interested in different life stages (e.g., Parrotfish) were visually determined to be in either their juvenile/initial phase or terminal (adult) phase.

We also collated baseline data from previous monitoring effort using the Atlantic and Gulf Rapid Reef Assessment (AGRRA) protocol, identical to the described fish transects above, which took place along Grand Cayman harbour in July 2018 to compare fish communities before vs during and after lockdown. Fish point transects followed the same protocol, with biomass and trophic guild calculations as above. However only AGRRA fish species^55^ are collected during fish transects when using the AGRRA protocol, therefore comparison before-during-after lockdown were only made using the AGRRA fish species^55^. The sites from 2018 AGRRA surveying were Cemetery (19.362667, − 81.398083), Happs Pipeline (19.38538, − 81.41645), and Sunset (19.2863, − 81.391417). While these sites are different to the sites surveyed during lockdown, they are part of the same reef system. However, these sites should experience less anthropogenic stress from human activities as they are all further away from where cruise ships moor compared to sites surveyed during COVID. Thus, using these three sites as a baseline for how the fish community changed is a conservative approach, as they are under relatively less stress compared to the site surveyed during lockdown.

### Statistical analysis

To assess the change in reef fish species richness, biomass, and abundance since lockdown, we implemented multivariate random effect Bayesian models parameterised with a negative binomial distribution. We selected a negative-binomial family distribution as the responses of species richness and abundance represent count data, while biomass can be rounded to an integer with no discernible influence on the outcome. Additionally, Negative-Binomial is preferable to a Poisson for these data because all three responses were over-dispersed based on preliminary analysis with residual deviance exceeding degrees of freedom^56^. We also explored non-linear and Gaussian models on raw and log transformed responses, but found model fit was consistently poor^57^ based on posterior predictive checks (PPC) and weighted alternative information criteria (WAIC). The three response variables were not statistically colinear based on a Spearman's rank test of collinearity using a conservative 0.65 threshold, nor was the predictor of rescaled dates. Dates were rescaled to represent integers, starting from zero, which represents the time of the first survey and so on sequentially. Our model also included the random effect of site against the intercept to control for spatial variation in fish communities across reefs^58^. We ran models for all fishes in the dataset, and again for each trophic guild separately. We also developed a univariate Bayesian random effect model to specifically determine the change in all parrotfish, and juvenile parrotfish biomass over the study period. Models were specified with flat uninformative priors for the fixed effect of date (rescaled), a student-t with shape 3 location 9.3 and scale 2.5 for the intercept which allows for flexibility in estimation while constraining within a reasonable range, and a weakly informative prior for the random effect of site with shape 3 location 0 and scale 2.5. The shape parameter of the responses was specified with a gamma prior (shape = 0.01, rate = 0.01) allowing for more heavy-tailed distributions. All models ran with 3000 iterations and 1500 warmups split across 4 chains using the “brms” package^59^, which uses STAN to develop flexible Bayesian models^60^ in R 4.3^61^. All models were inspected for convergence using visual inference of trace plots and considered to converge when the Rhat value (Gelman Rubin statistic) equalled one^59^. Model fit was confirmed through visual inspection of posterior predictive checks for each response variable.

To statistically compare fish biomass before, during, and after COVID lockdown we used Kruskal–Wallis test on the three grouping periods as data were not normally distributed based on visual inference of histograms and a Shapiro-Wilks test of normality. Post-hoc comparisons were carried out using a Dunn's test from the FSA package^62^ with p-values adjusted using the Holm's method. Community composition of fish was compared using non-Metric Multi-Dimensional Scaling (nMDS) in the vegan package^63^. The nMDS community matrix was square-root transformed and subsequently transformed into a Bray–Curtis dissimilarity matrix. The dissimilarity matrix was used to statistically compare time periods of the fish communities using a PERMANOVA from the vegan package. Additionally, we conducted a pairwise PERMANOVA using the pairwiseAdonis package^64^.

### Supplementary Information

Supplementary Information.

## Data Availability

Data and code are available on out Github (https://github.com/JackVJohnson/Quiet-Oceans-Grand-Cayman).
