# Supplementary material for: Insights from the 2-year-long human confinement experiment in Grand Cayman reveal the resilience of coral reef fish communities
Source: Sci Rep. 2023 Dec 9;13:21806. doi: 10.1038/s41598-023-49221-y (PMC10710434; doi:10.1038/s41598-023-49221-y)
Supplement: Supplementary file 1 — Supplementary Information. [file 41598_2023_49221_MOESM1_ESM.docx]

***Supplementary information for:***

**Insights from the two-year-long Human Confinement Experiment in Grand Cayman reveal the resilience of coral reef fish communities**

Jack V. Johnson^1^, Alex D. Chequer^1^, Gretchen Goodbody-Gringley^1^

^1^Reef Ecology and Evolution Lab, Central Caribbean Marine Institute, Little Cayman, Cayman Islands

Corresponding Author: [jackvjohnson@hotmail.com](mailto:jackvjohnson@hotmail.com)


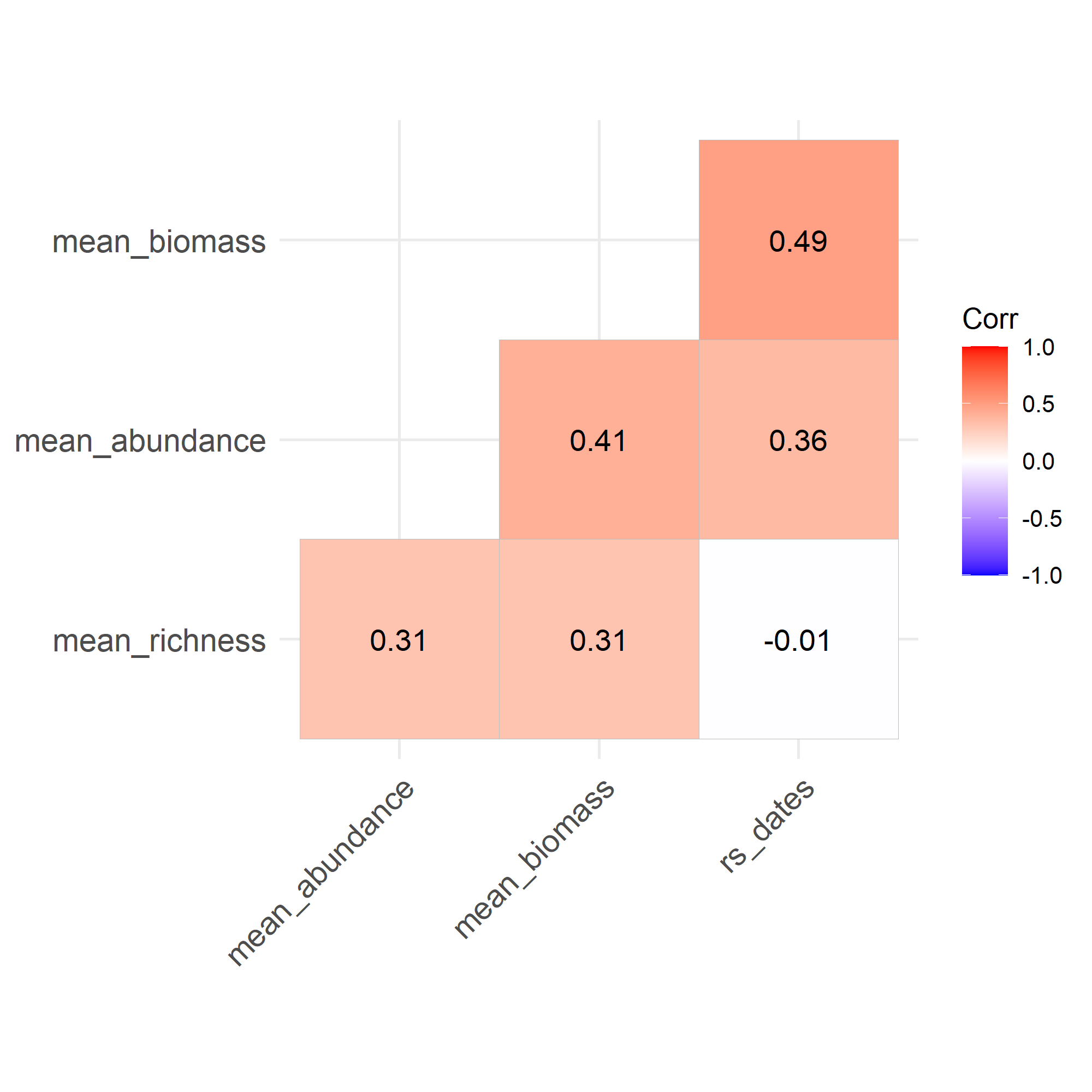


**Fig S1.** Correlation matrix of response variables and predictor used in the Bayesian models based on all fish data. None of the variables show high levels of collinearity (>.65).

**Table S1.** Overview of trophic and taxonomic composition (abundance and species richness) used in the study.

| **Trophic guild** | **Family** | **Total abundance** | **Number of species** |
| --- | --- | --- | --- |
| Herbivore | Scaridae | 12968 | 19 |
| Herbivore | Acanthuridae | 75 | 1 |
| Invertivore | Serranidae | 606 | 9 |
| Invertivore | Haemulidae | 477 | 7 |
| Invertivore | Holocentridae | 533 | 5 |
| Invertivore | Chaetodontidae | 500 | 4 |
| Invertivore | Labridae | 1592 | 4 |
| Invertivore | Sparidae | 23 | 3 |
| Invertivore | Diodontidae | 14 | 2 |
| Invertivore | Mullidae | 157 | 2 |
| Invertivore | Ostraciidae | 15 | 2 |
| Invertivore | Pomacanthidae | 157 | 2 |
| Invertivore | Apogonidae | 3 | 1 |
| Invertivore | Carangidae | 391 | 1 |
| Invertivore | Chaenopsidae | 1 | 1 |
| Invertivore | Cirrhitidae | 6 | 1 |
| Invertivore | Echeneidae | 1 | 1 |
| Invertivore | Gerreidae | 28 | 1 |
| Invertivore | Labrisomidae | 16 | 1 |
| Invertivore | Monacanthidae | 2 | 1 |
| Invertivore | Pomacentridae | 12 | 1 |
| Invertivore | Synodontinae | 3 | 1 |
| Invertivore | Tetraodontidae | 9 | 1 |
| Macrocarnivore | Lutjanidae | 450 | 9 |
| Macrocarnivore | Serranidae | 423 | 8 |
| Macrocarnivore | Muraenidae | 127 | 3 |
| Macrocarnivore | Carangidae | 6 | 2 |
| Macrocarnivore | Scorpaenidae | 24 | 2 |
| Macrocarnivore | Aulostomidae | 26 | 1 |
| Macrocarnivore | Bothidae | 1 | 1 |
| Macrocarnivore | Dasyatidae | 4 | 1 |
| Macrocarnivore | Ginglymostomatidae | 1 | 1 |
| Macrocarnivore | Megalopidae | 36 | 1 |
| Macrocarnivore | Priacanthidae | 12 | 1 |
| Macrocarnivore | Sciaenidae | 7 | 1 |
| Macrocarnivore | Scombridae | 19 | 1 |
| Macrocarnivore | Sphyraenidae | 14 | 1 |
| Macrocarnivore | labridae | 9 | 1 |
| Omnivore | Pomacentridae | 15409 | 8 |
| Omnivore | Gobiidae | 1518 | 5 |
| Omnivore | Acanthuridae | 4306 | 2 |
| Omnivore | Grammatidae | 3772 | 2 |
| Omnivore | Labridae | 12003 | 2 |
| Omnivore | Monacanthidae | 21 | 2 |
| Omnivore | Pomacanthidae | 39 | 2 |
| Omnivore | Kyphosidae | 363 | 1 |
| Omnivore | Serranidae | 27 | 1 |
| Omnivore | Tetraodontidae | 1453 | 1 |
| Planktivore | Balistidae | 41 | 2 |
| Planktivore | Pomacentridae | 17339 | 2 |
| Planktivore | Holocentridae | 23 | 1 |
| Planktivore | Labridae | 6 | 1 |


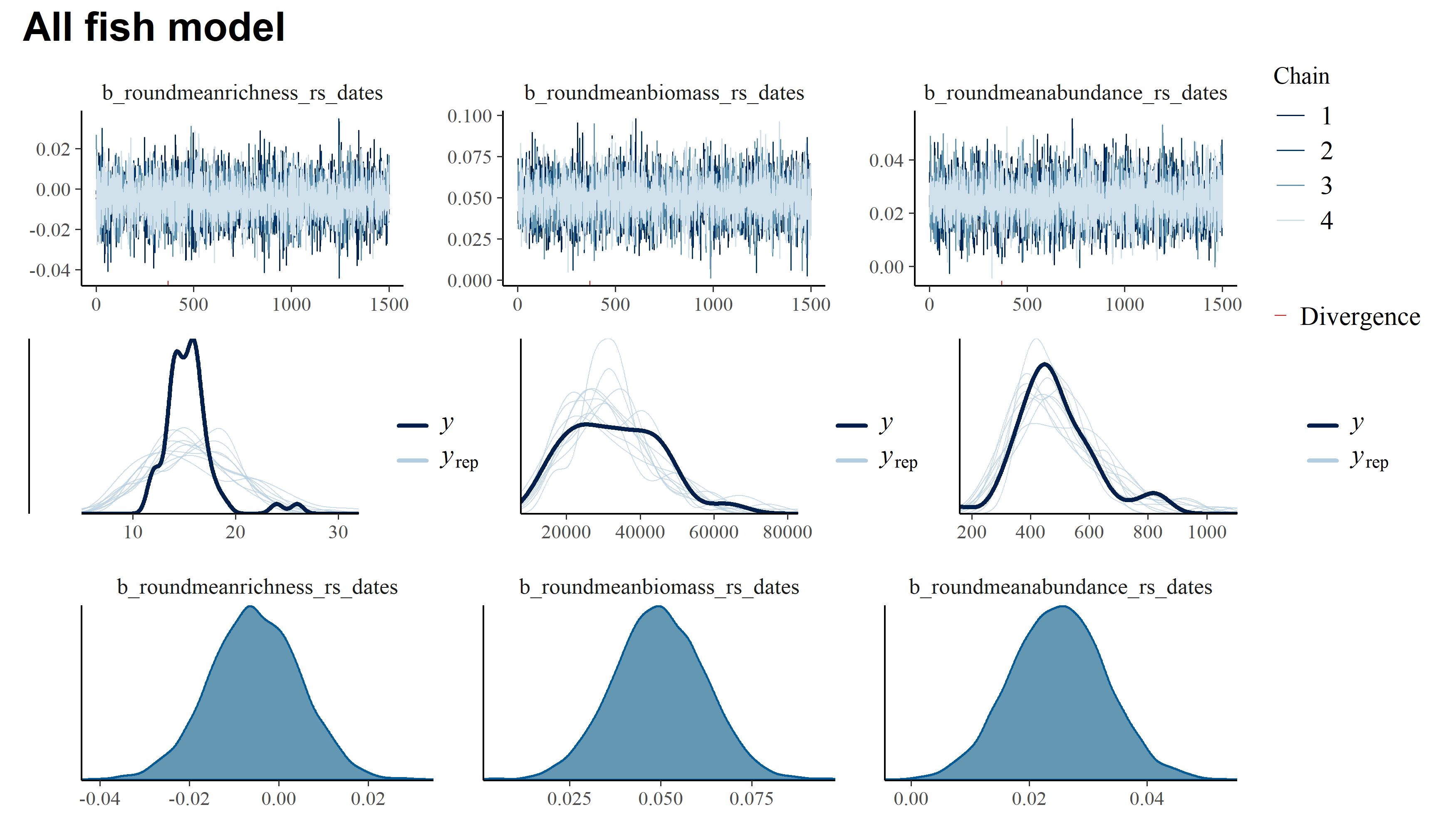


**Fig S2**


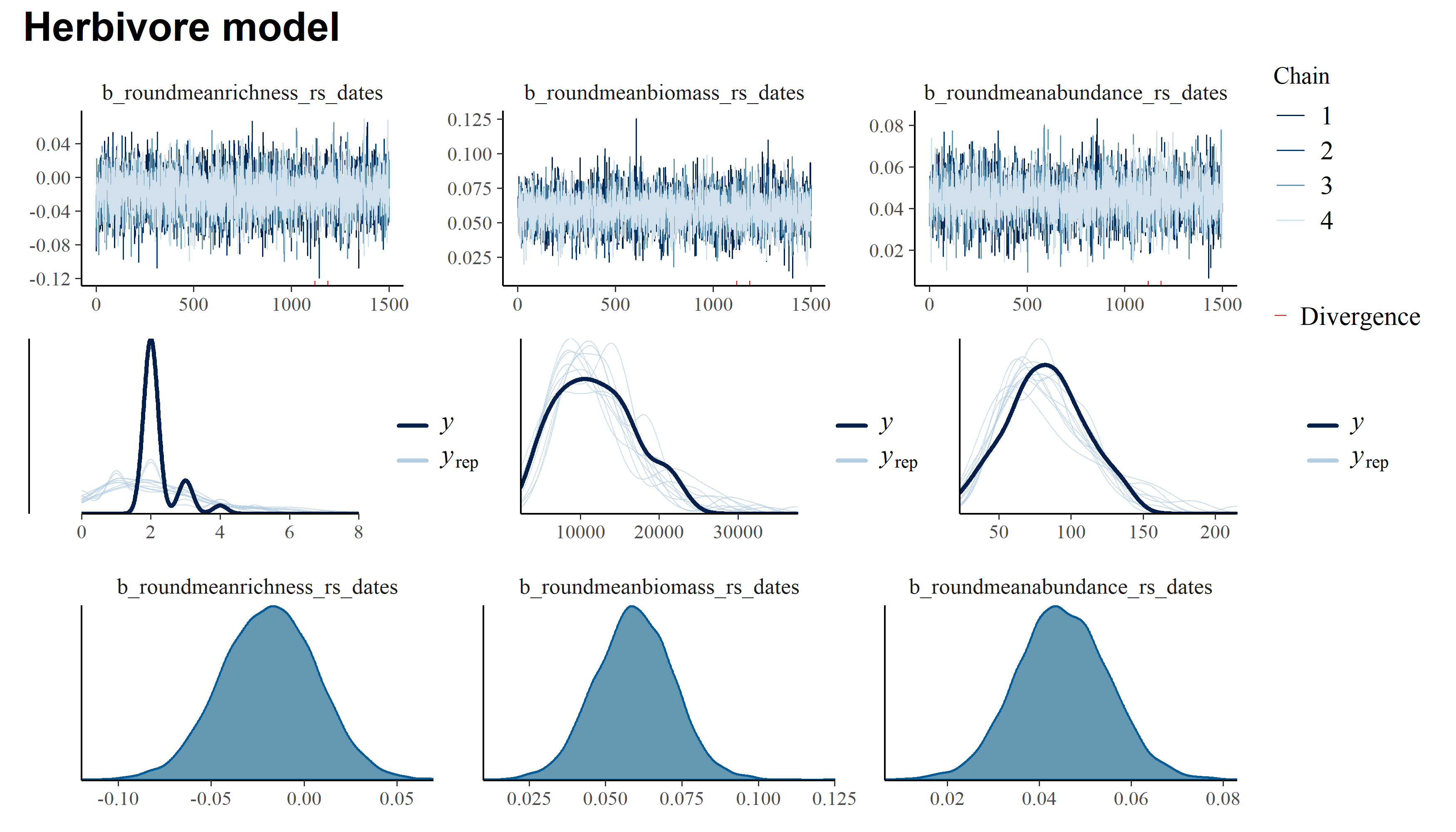


**Fig S3**


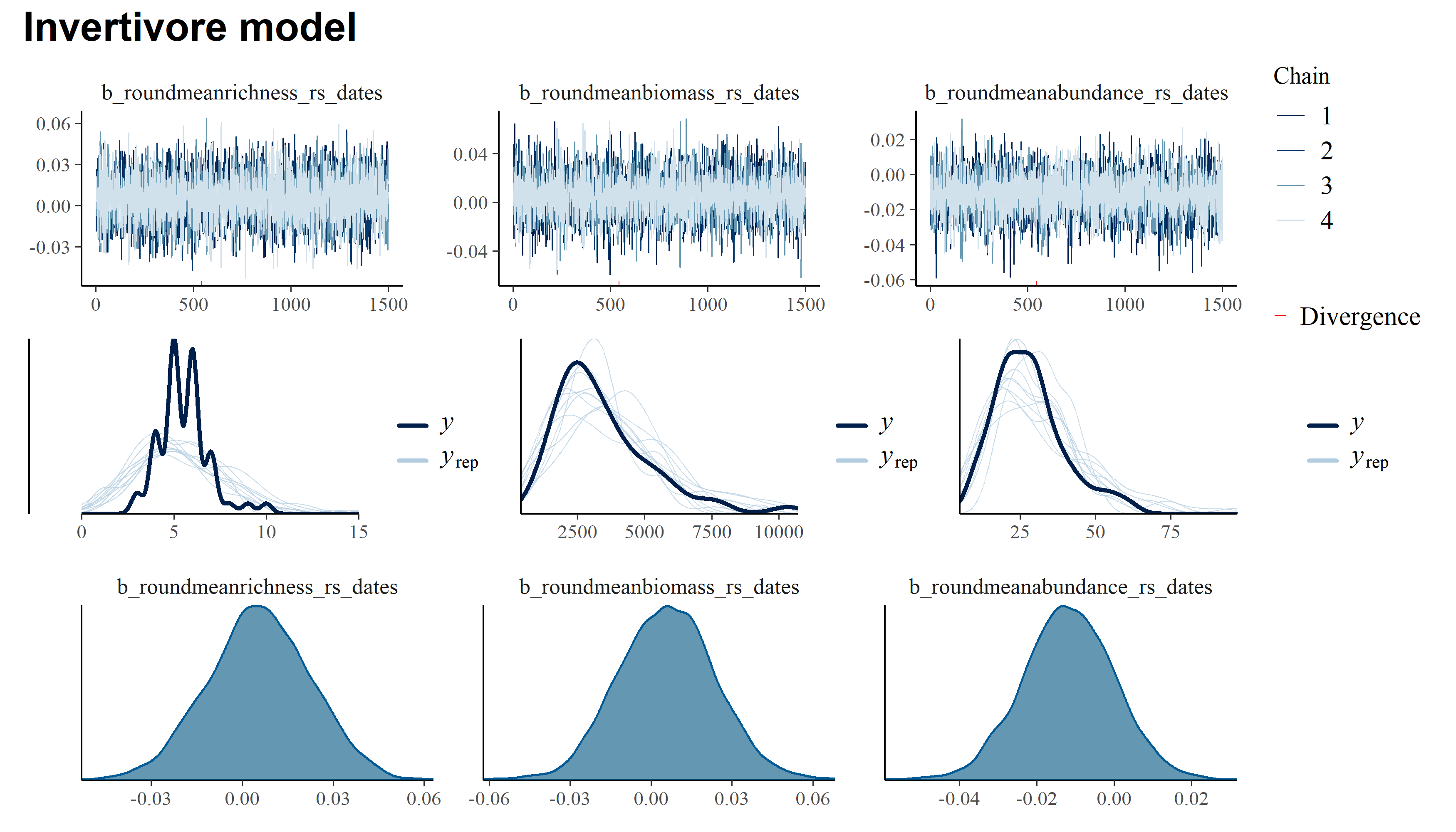


**Fig S4**


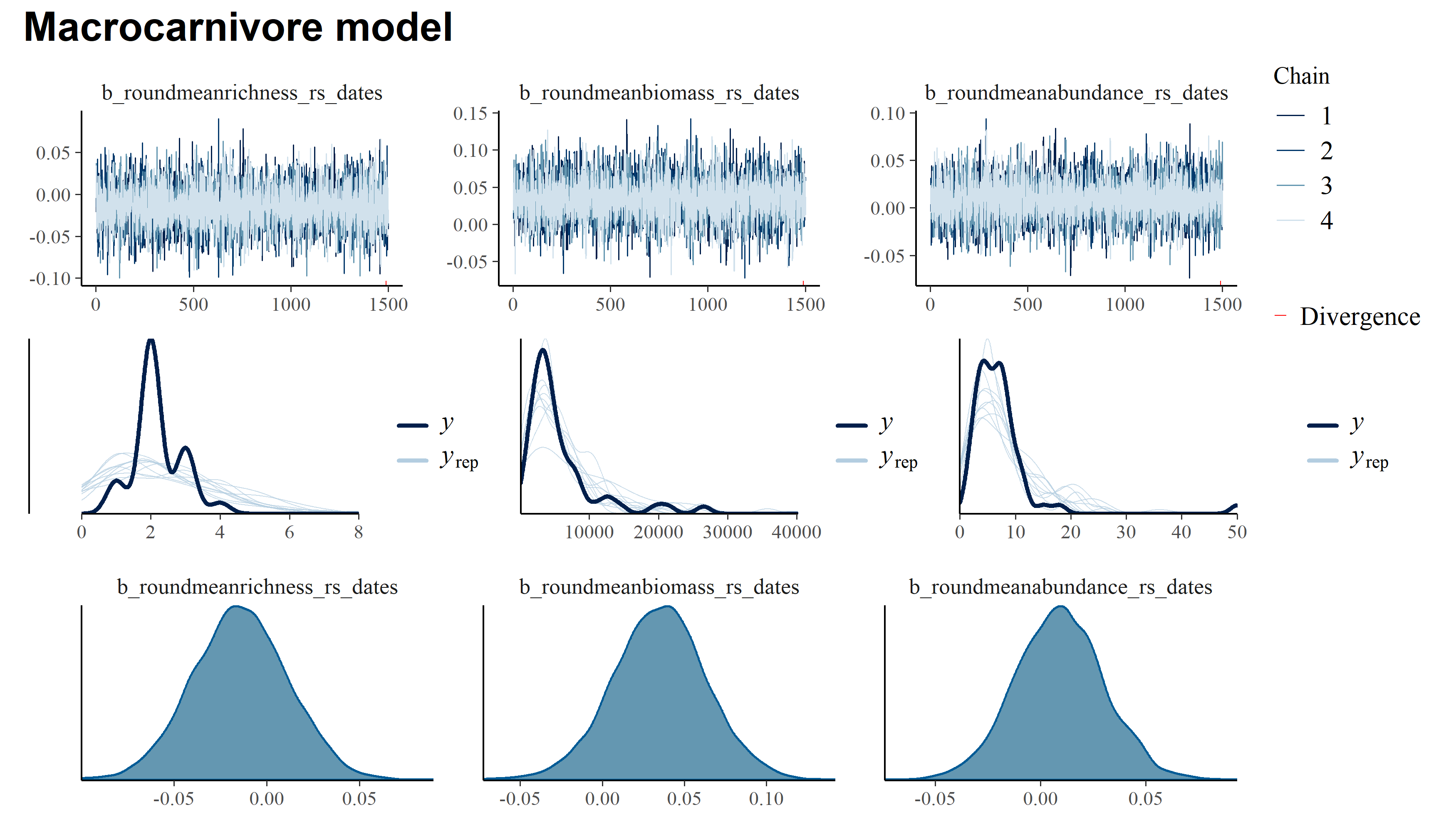


**Fig S5**


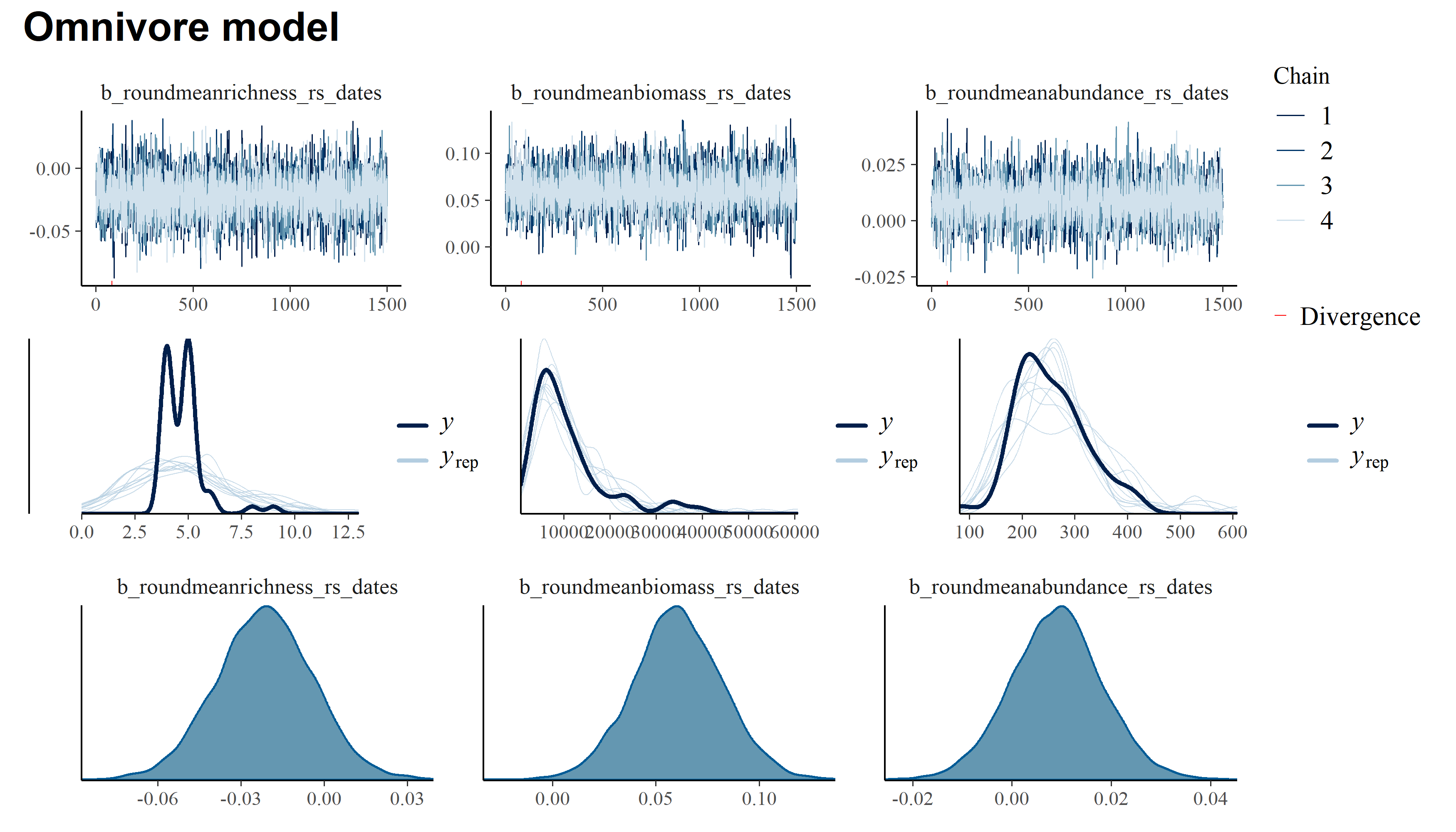


**Fig S6**


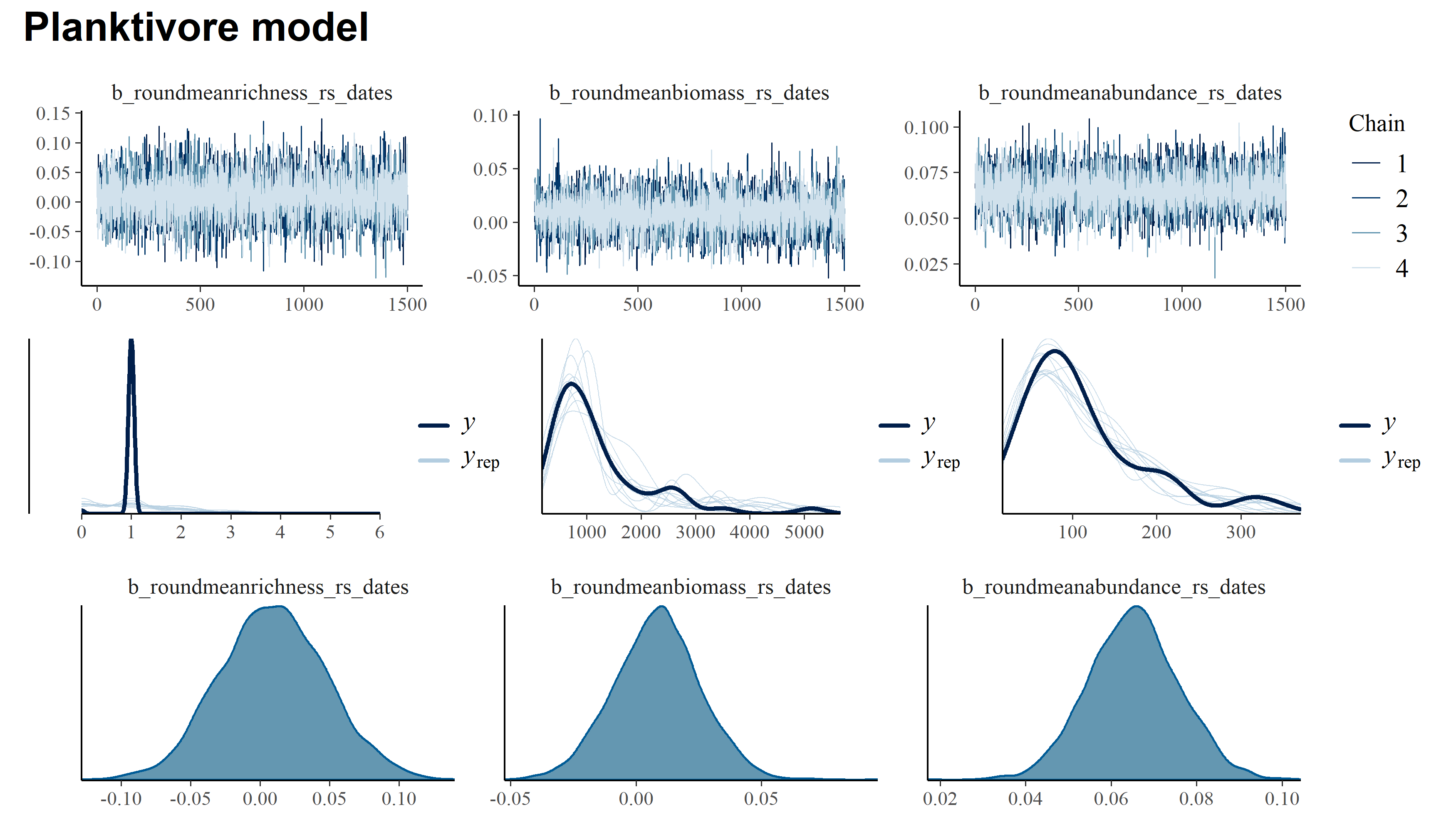


**Fig S7**


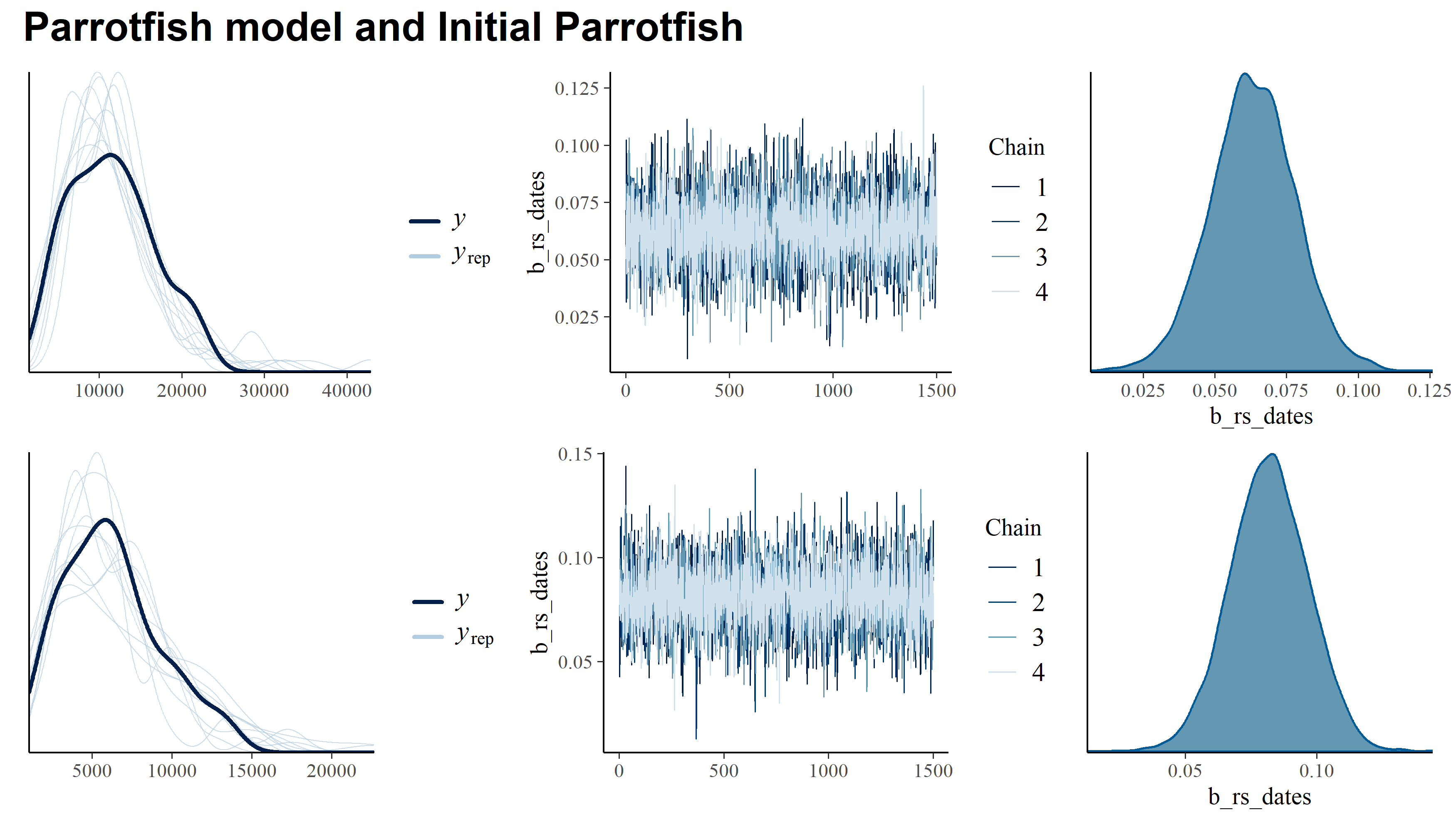


**Fig S8**

**Figs S2 – S8.** Trace plots, posterior predictive checks, and posterior distributions from Bayesian models. All models are denoted by their accompanying titles.

**Supplementary methods**

To provide an overview of changes in the species composition before lockdown (2018 AGRRA sampling) and during lockdown, we performed a negative-binomial family Generalised Linear Model (GLM) on the raw count data for species abundances before compared to during lockdown using the ‘manyabund’ package in R ^1^. A negative-binomial family distribution was used because model performance is often better than a Poisson distribution-based model ^2^. From the ‘manyglm’ model, we conducted a ‘anova.manyglm’ using a Wald test score to compute test and p-values for the effect differences in fish abundance before compared to during lockdown. All test scores are displayed in Table S2. Full code used can be found: <https://github.com/JackVJohnson/Quiet-Oceans-Grand-Cayman/tree/main>.

**Table S2**. Output from the anova.manyglm computed in the mvabund package for abundances for fish abundances of each species before compared to during lockdown. Only fish species with sufficient samples across periods and high enough abundances were included in the test.

|  | **P value** | **Wald test score** |
| --- | --- | --- |
| Acanthurus_coeruleus | 0.020 | 69.071 |
| Scarus_iseri | 0.020 | 34.728 |
| Sparisoma_aurofrenatum | 0.020 | 61.632 |
| Sparisoma_viride | 0.020 | 88.269 |
| Caranx_ruber | 0.020 | 27.809 |
| Lutjanus_apodus | 0.020 | 36.003 |
| Scarus_taeniopterus | 0.020 | 67.408 |
| Bodianus_rufus | 0.020 | 27.146 |
| Haemulon_carbonarium | 0.020 | 15.563 |
| Stegastes_planifrons | 0.020 | 141.577 |
| Cephalopholis_cruentata | 0.039 | 12.648 |
| Microspathodon_chrysurus | 0.039 | 13.559 |
| Pomacanthus_paru | 0.059 | 11.118 |
| Haemulon_parra | 0.098 | 8.251 |
| Chaetodon_ocellatus | 0.098 | 9.304 |
| Cephalopholis_fulva | 0.157 | 7.827 |
| Scarus_vetula | 0.275 | 6.879 |
| Mycteroperca_tigris | 0.392 | 5.895 |
| Chaetodon_capistratus | 0.588 | 5.456 |
| Haemulon_flavolineatum | 0.686 | 3.962 |
| Haemulon_plumierii | 0.686 | 4.588 |
| Ocyurus_chrysurus | 0.686 | 4.777 |
| Sparisoma_atomarium | 0.686 | 4.505 |
| Lutjanus_mahogoni | 0.686 | 4.029 |
| Pterois_volitans | 0.686 | 4.626 |
| Epinephelus_guttatus | 0.686 | 4.894 |
| Chaetodon_striatus | 0.843 | 3.445 |
| Cantherhines_macrocerus | 0.863 | 2.873 |
| Sparisoma_rubripinne | 0.863 | 2.759 |
| Haemulon_sciurus | 0.863 | 2.876 |
| Melichthys_niger | 0.863 | 2.077 |
| Diodon_holocanthus | 0.863 | 2.858 |
| Lachnolaimus_maximus | 0.863 | 2.776 |
| Epinephelus_adscensionis | 0.863 | 2.847 |
| Scarus_coelestinus | 0.941 | 1.986 |
| Acanthurus_tractus | 1.000 | 0.092 |
| Sparisoma_chrysopterum | 1.000 | 1.458 |
| Calamus_calamus | 1.000 | 1.792 |
| Lutjanus_jocu | 1.000 | 0.453 |
| Sphyraena_barracuda | 1.000 | 0.492 |
| Lutjanus_analis | 1.000 | 0.652 |
| Epinephelus_striatus | 1.000 | 0.000 |
| Sphoeroides_spengleri | 1.000 | 0.000 |
| Acanthurus_chirurgus | 1.000 | 0.937 |
| Scarus_coeruleus | 1.000 | 0.000 |
| Halichoeres_radiatus | 1.000 | 1.898 |
| Prognathodes_aculeatus | 1.000 | 0.000 |
| Diodon_hystrix | 1.000 | 0.000 |
| Cantherhines_pullus | 1.000 | 1.898 |
| Haemulon_album | 1.000 | 0.000 |
| Pomacanthus_arcuatus | 1.000 | 0.000 |
| Lutjanus_griseus | 1.000 | 0.925 |
| Lutjanus_synagris | 1.000 | 0.925 |
| Mycteroperca_bonaci | 1.000 | 0.925 |
| Mycteroperca_interstitialis | 1.000 | 0.000 |
| Canthidermis_sufflamen | 1.000 | 0.925 |
| Aluterus_scriptus | 1.000 | 0.000 |
| Trachinotus_falcatus | 1.000 | 0.937 |
| Scarus_guacamaia | 1.000 | 1.850 |
| Calamus_bajonado | 1.000 | 0.000 |
| Calamus_pennatula | 1.000 | 0.925 |
| Anisotremus_surinamensis | 1.000 | 0.925 |


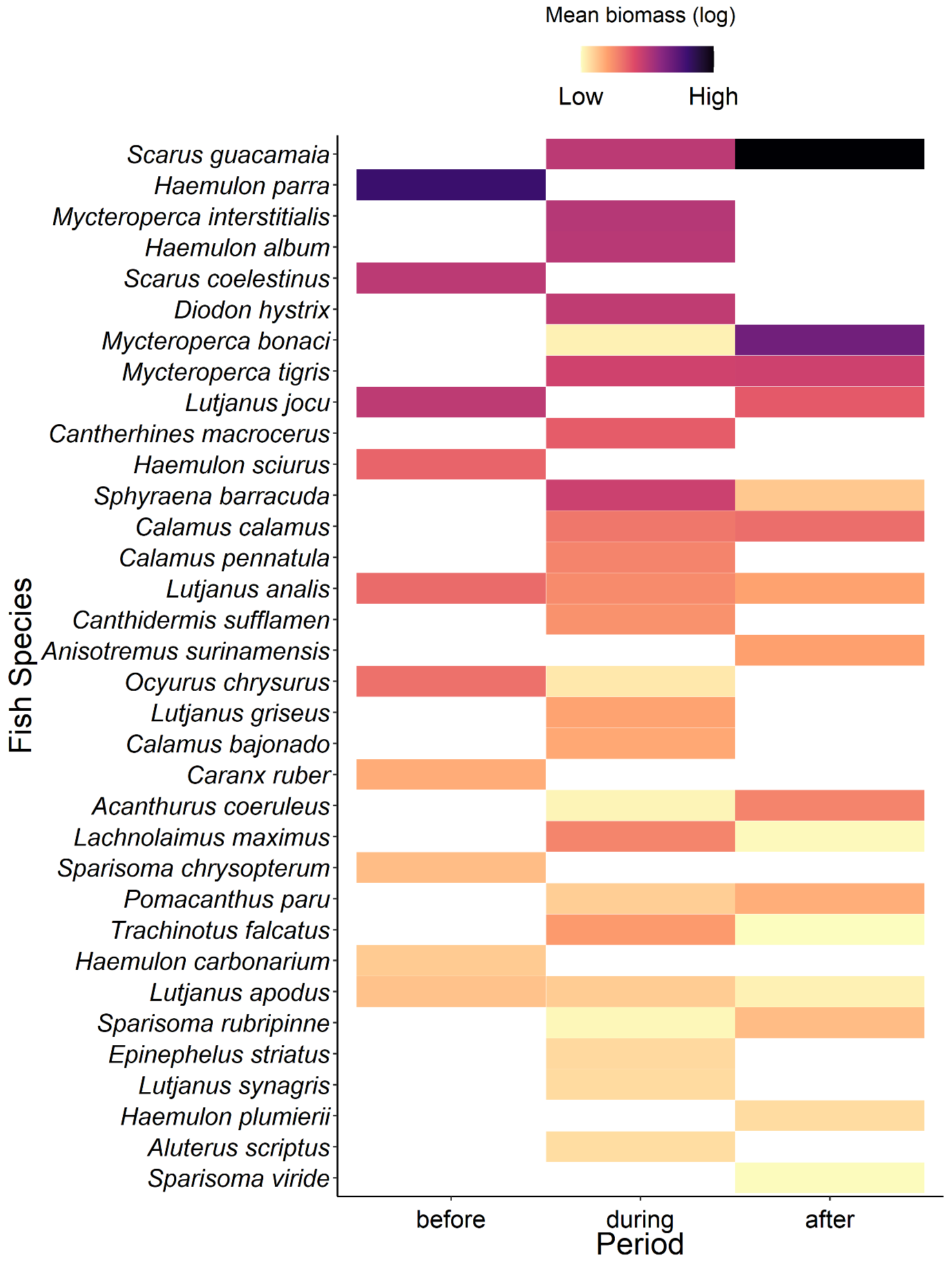


**Fig S9**. Relative mean biomass (log) for fish species across sampling periods (before, during, and after lockdown).

**Supplementary references**

1. Wang, Y., Naumann, U., Wright, S. T. & Warton, D. I. mvabund– an R package for model-based analysis of multivariate abundance data. *Methods Ecol. Evol.* **3**, 471–474 (2012).

2. Warton, D. I. Many zeros does not mean zero inflation: comparing the goodness-of-fit of parametric models to multivariate abundance data. *Environmetrics* **16**, 275–289 (2005).
